# Harris on Air Chamber

**Published:** 1855-04

**Authors:** John Harris

**Affiliations:** Salem O


### AIR CHAMBERS.

Mr. Editor:—As I am not entirely decided in my own mind, in relation to the propriety of air chambers for retaining upper sets of teeth. I should be glad that you, or some of your contributors would favor us with some animadversions upon the subject in the next number of the Register. In my practice, I have made perhaps about an equal number of each kind, that is with and without air chambers. Some of my very most successful cases were without chambers; and such *adaptation* of the plate, as does not require it to contain a chamber, or to be very wide in the arch of the mouth, seems to me a desideratum devoutly to be wished for. And yet when I observe that nearly all the upper sets, made by other dentists, which have lately come under my observation, contain chambers, I feel distrustful of my own predilection for plates without them. In the use of air chambers, I have for some time past been very much governed by the depth of the alveolar ridge, and the favorableness of the antagonism of the opposite teeth. When there is sufficient depth of process combined with favorable antagonism, I dispense with chambers. When an opposite condition of process, and opposing teeth exists I generally use them, proportionate in size with the relative condition of the month. According to my experience, plates containing chambers frequently adhere better for a short time after worn than subsequently, while many plates without chambers continue to improve in their adhesive quality for some time after worn.

Am I mistaken in the conclusion I have come to, from what I have seen of the work of others, that most dentists at the present time use air-chambers in nearly all their plates for upper sets? What is your own practice in this respect, at the present time, and why? Has a narrow plate without any chamber that adheres well, any advantages over a wide plate covering the whole roof of the mouth, with a chamber setting down from it, that adheres equally well.

SALEM O, FEB. 14th, 1855. JOHN HARRIS.

In reply to the inquiry of Dr. Harris, we would say that we use air chambers in a large majority of cases, with a tolerably wide plate for upper sets. A plain plate which holds sufficiently firm, we, however, regard as preferable, and when this can be accomplished and the plate not cover much of the palate, it is still better. It is more pleasant to the wearer not to have the entire palate covered with a metalic substance. The fact that plain plates increase in adhesion more than air chamber plates can be accounted for, from the fact that unless the chamber is very large, it fills up more or less by the soft parts adapting themselves to the vacuum, and thus rendering the chamber useless. The plain plate is improved because the adaptation becomes more perfect by use.

We are now inserting an upper set without an air chamber, because the palital arch is almost a flat surface, and the chamber would interfere with the articulation. In this case, our plain plate adheres better than the one before used which has a chamber.

Plain plates which do not adhere sufficiently tight, we change to those with chambers. We scarcely ever make a chamber other than that we get in swedging the plate, with the pattern of the chamber on the metallic model. We regard the accurate adaptation of the plate around the external alveolar border as of the greatest importance, and the plate should extend up as high as the soft parts will permit. The better this is ac. complished, the less will the palital arch have to be covered. Many plates, we are satisfied, cover too much of the soft palate and are rendered less useful by it, for the moveability of these parts in deglution often loosen the plate, when otherwise it would retain its place. The great desideratum in suction plates should be not so much the force of adhesion as the stability of the plate, when pressure is made on it at different points.

A plate which will not loose its hold by pressure upon it at any point, and yet can be drawn from the mouth by little force is far better than one requiring ten or fifteen pounds force to remove it, and which will loose its hold by slight pressure on the front teeth or on either side.—[Ed.

